# Obesity in primary care: prevention, management and the paradox

**DOI:** 10.1186/1741-7015-12-149

**Published:** 2014-08-26

**Authors:** David Haslam

**Affiliations:** GP Watton at Stone, Hertfordshire, Centre for Obesity research, Luton & Dunstable Hospital, Bedfordshire, UK

**Keywords:** Obesity, Primary care, Obesity paradox, Obesity management, Weight

## Abstract

Government and societal efforts to combat obesity are aimed at prevention, although there is a generation for whom excess weight is the rule rather than the exception. Although measures to prevent a worsening of the current epidemic are important, management of obesity must also be prioritised. Obesity management is beset with problems ranging from attitudinal to clinical and pharmacological, and the individualisation of therapy.

## Background

Obesity prevention has failed. If nobody in the UK gains another single ounce, there are enough already obese people to make epidemics of diabetes, then heart disease, then premature death inevitable. The job of Primary Care is to manage obesity, although this may not involve the loss of a single ounce: their role is often misunderstood, especially by departmental and Government bodies, and especially by the Quality and Outcomes Framework (QOF) which perversely incentivises maintaining excess weight in order to bulk up the obesity register without a finger being lifted to introduce screening or management of the condition.

## Prevention

However, three-quarters of the population have avoided obesity – although more than half of those are overweight - so measures must be taken to prevent weight gain in the public health environment. The National Obesity Forum behavioural expert Damian Edwards undertook a social experiment in a Salford supermarket, involving measures such as rearranging shelves to make healthy food more accessible and placing life-size cut outs of smiling local general practitioners (GPs) and nurses indicating the fruit and vegetables. These simple changes carried out overnight, increased fruit consumption by almost 30% compared to control [1]. This, and other simple changes, such as removing sweets from the checkout, and changing the GP incentive QOF to reward obesity management, would have an instant beneficial effect on the health of the Nation. Other measures will take longer – the Action on Sugar campaign will specify targets for food reformulation, but will take several years to make a big difference; changes to the built environment to promote activity might take a generation to have an effect. Industry, public health and Government have a major role to play, and the political power wielded by the food and retail industries may thwart change. Other proven determinants of obesity, such as genetic and epigenetic influences, gut microbiota and adenovirus infections, may never alter.

## Management

Primary care bears the brunt of obesity management; obese patients may present for weight loss advice, may attend clinics such as diabetes or cardiovascular disease (CVD) clinics as a secondary problem to their weight, or, more likely, will attend with something entirely unrelated to their weight, which could be as diverse as holiday jabs to a black eye. The unique challenge for the GP or nurse is to engage the latter group effectively and inoffensively, a conversation which represents the start of the obesity management programme. This process may only take the last two minutes of an unrelated consultation – engagement occurs, weight and blood pressure are measured, blood tests organised and more comprehensive follow-up assured. Thus, the initial phase of weight management is the assessment of baseline characteristics and the rapid correction of possible features, such as hypertension, dyslipidaemia or type 2 diabetes. Other common co-existing conditions which may not have presented as symptoms in their own right and can easily be screened for, include sleep apnoea, nonalcoholic steatohepatitis (NASH) and polycystic ovarian syndrome.

The remaining aspect of obesity management is attempting to induce weight loss and maintenance. Major weight loss is difficult to induce in primary care as demonstrated by studies such as Counterweight [2] and Camwel [3] which struggled to induce and maintain meaningful weight loss. On the other hand, the Look Ahead study [4] showed that long term weight loss is sustainable, linked with improvements in lipids and blood pressure, but in an intervention too intensive to be transferable to primary care. Similarly, the Diabetes Prevention Programme [5] and the Diabetes Prevention Study [6], although similarly untransferable to routine community interventions, show that remarkably small degrees of weight loss – less than a kilogramme per year – confer 58% reduction (in both studies) in the cumulative incidence of diabetes.

Cornerstones of obesity management in primary care are diet, physical activity and behavioural therapy. Low-carbohydrate diets are increasing in popularity following studies, such as A-to-Z [7], which demonstrate their effectiveness. Exercise on prescription has limited evidence for benefit, and building activity into routine daily life is important [8] although scheduled exercise does have cardiometabolic benefit. Behavioural therapy involves elements such as goal setting, stimulus control, cognitive restructuring and relapse prevention, which tend naturally to be done as part of the conversation on diet and physical activity.

## Pharmacotherapy

Pharmacotherapy for obesity is restricted to orlistat, which induces weight loss by blocking the action of pancreatic and gastric lipase, reducing absorption of fat by up to one-third. The unabsorbed dietary fat is excreted through the intestine. More importantly, it reduces the cumulative incidence of diabetes and has been shown to reduce total and LDL cholesterol and increase HDL:LDL ratio [9].

Sibutramine is a centrally acting satiety enhancer, which was popular, effective and well-tolerated prior to its withdrawal in 2010. The SCOUT study [10] showed an increase in non-fatal CV events in patients with already existing diabetes and CVD but had no stopping criteria, so non-responders continued the drug for five times longer than the clinical licence allowed. The sibutramine was withdrawn on the grounds that any obese person could have latent CVD; however, a subsequent sub analysis [11] proved that had the licence been followed, even in these high risk patients, and non-responders taken off the drug, then mortality was reduced. Furthermore, two anti-obesity drugs authorised in the US by the Food and Drug Administration (FDA) – Qsymia and Belviq - were rejected in Europe on the spurious grounds that they might be used for reasons other than those for which they were intended. A third – Contrave – was delayed by the FDA awaiting CV outcome studies. In treating obesity in the context of serious chronic disease, clinicians require licensing authorities to look at the benefits of obesity reduction in treating and preventing diabetes, CVD, NASH and so on. Taking into account the controversial withdrawal of rimonabant in 2008, there are five times as many drugs withdrawn or remaining unlicensed, than there are drugs available for prescription. Liraglutide, an injectable glucagon-like peptide 1 (GLP-1) agonist already being used to treat diabetes, has been filed at a higher dose for the management of obesity [12].

Primary care is also where most of the bariatric surgery programme takes place – everything from engagement to death, apart from the brief technological interlude of surgery and immediate follow-up.

In managing obesity and its associated risk factors and co-morbidities, clinicians encounter problems, as managing one individual element may exacerbate another. In particular, glucose-lowering agents, specifically insulin, sulphonylureas and thiazoledinediones have had the demoralising and unhealthy side effect of weight gain. Both β-blockers and thiazide diuretics increase the risk of diabetes; sibutramine induced weight loss but raised blood pressure; statins improve lipid profile but increase the risk of diabetes; niacin increases HbA1c whilst lowering cholesterol; torcetrapib was withdrawn from phase 3 trials despite enormous improvements in lipid profile because of an increase in blood pressure and stroke risk; β-blockers reduce blood pressure but increase the risk of obesity, partly by inducing more sedentary behaviour. Progress has been made with the advent of DPP-4 inhibitors, GLP-1 agonists and SGLT-2 inhibitors: glucose lowering agents which are weight neutral or induce weight loss and have various other beneficial effects on cardiometabolic parameters.

## The obesity paradox

Although a precursor of cardiometabolic diseases and cancer [13], the presence of obesity may protect against mortality once these conditions have occurred. It has been commented that ‘the idea that a known risk factor somehow transforms into a ‘protective’ agent after an occurrence of a vascular clinical event is both surreal and troubling’ [14]. This effect is termed the ‘Obesity Paradox’. Increased body mass index (BMI) is a determinant for heart failure (HF): for every 1 unit increase in BMI, risk of HF increases by 5% in men and 7% in women. However, once HF has occurred the opposite is true; a meta-analysis of 28,209 recruits [15] showed a reduction in CV mortality of 40% and all-cause mortality of 33%. In a review of trials including 250,000 individuals with coronary artery disease, cardiovascular and mortality outcomes were better in overweight and ‘mildly’ obese patients compared with ‘normal’ weight [16]. The INVEST [17] study included 22,500 individuals with hypertension and coronary artery disease, and demonstrated a lower mortality and major CV events in the overweight and obese compared to those of normal weight. Various reasons have been postulated for the paradox: fat may genuinely exert a protective influence through unknown mechanisms, possibly through improved metabolic reserve, or obesity may lead to individuals becoming identified earlier as high risk, and treated earlier and more vigorously with antihypertensive agents and statins. An interesting thesis suggests that individuals who only had HF because of weight gain, are naturally less susceptible to the disease and, therefore, have a more favourable prognosis [18]. Others suggest that lower weight might be due to intercurrent illness or be smoking-related, or that BMI is used inappropriately as a measure of adiposity, although recent studies adjusted for these factors [13]. A *post-hoc* analysis of the PROactive study of pioglitazone assessed the obesity paradox, with interesting results [19]. In patients with type 2 diabetes mellitus (T2DM) and CVD, the lowest mortality was with BMI 30 to 35, whereas those with BMI <22 had higher all-cause mortality. Active weight loss was associated with increased mortality and hospitalisation: loss of ≥7.5% body weight (seen in 18.3% of patients) was the strongest predictor of impaired survival whereas weight gain was not associated with increased mortality. The positive relationship between obesity and mortality is attenuated with age, implying that excess weight may act as a protective factor in established chronic disease [20].

## Conclusions

Nobody attends their GP asking for their obesity to be prevented; obesity has occurred by the time the health care professional becomes involved. The most important point in the obesity management programme is the initial engagement of the patient followed by screening and risk management. Weight loss is appropriate and should be encouraged in many patients, but individualisation of care is important so that the wrong patients do not have weight loss induced, and those who would benefit from maintaining weight are properly identified. Prevention of obesity and, therefore, chronic disease is the domain of Government, public health and industry and should be urgently prioritised.

## Author information

DH is a full time GP with a special interest in obesity and cardiometabolic disease, Physician in Obesity Management, Luton & Dunstable Hospital, and Chair of the National Obesity Forum (NOF). The NOF is a charity whose aim is to increase awareness of obesity, diabetes and weight related illness, and improve their management. It provides educational material for clinicians, and also sits upon the Department of Health Obesity Strategy Review Group. He is Visiting Professor, Robert Gordon University, Aberdeen; Visiting Professor Chester University, and is the Obesity Specialist at 76 Harley St. He is a Board Member of ESCO (Experts on Severe and Complex Obesity) and of the charity Foundations, a Director of PCOS UK, a member of the Counterweight Board, and a Visiting Lecturer at Beds & Herts Postgraduate Medical School. DH speaks internationally on obesity and related diseases. His books include *Fat Gluttony and Sloth, Obesity in Art, Literature and Medicine*, and *The Obesity Epidemic, and its Management*, both published in 2010, and *Controversies in Obesity* and *Fast Facts: obesity* both published in 2014.
